# Glycogen storage disease type I: Genetic etiology, clinical manifestations, and conventional and gene therapies

**DOI:** 10.1002/pdi3.3

**Published:** 2023-07-24

**Authors:** Jiamin Zhong, Yannian Gou, Piao Zhao, Xiangyu Dong, Meichun Guo, Aohua Li, Ailing Hao, Hue H. Luu, Tong‐Chuan He, Russell R. Reid, Jiaming Fan

**Affiliations:** ^1^ Ministry of Education Key Laboratory of Diagnostic Medicine, and Department of Clinical Biochemistry School of Laboratory Medicine Chongqing Medical University Chongqing China; ^2^ Molecular Oncology Laboratory Department of Orthopaedic Surgery and Rehabilitation Medicine The University of Chicago Medical Center Chicago Illinois USA; ^3^ Department of Orthopedic Surgery The First Affiliated Hospital of Chongqing Medical University Chongqing China; ^4^ Laboratory of Craniofacial Biology and Development Department of Surgery Section of Plastic Surgery The University of Chicago Medical Center Chicago Illinois USA

**Keywords:** dietary therapy, drug therapy, gene therapy, glycogen storage disease type I, molecular genetics mechanism

## Abstract

Glycogen storage disease type I (GSDI) is an inherited metabolic disorder characterized by a deficiency of enzymes or proteins involved in glycogenolysis and gluconeogenesis, resulting in excessive intracellular glycogen accumulation. While GSDI is classified into four different subtypes based on molecular genetic variants, GSDIa accounts for approximately 80%. GSDIa and GSDIb are autosomal recessive disorders caused by deficiencies in glucose‐6‐phosphatase (G6Pase‐α) and glucose‐6‐phosphate‐transporter (G6PT), respectively. For the past 50 years, the care of patients with GSDI has been improved following elaborate dietary managements. GSDI patients currently receive dietary therapies that enable patients to improve hypoglycemia and alleviate early symptomatic signs of the disease. However, dietary therapies have many limitations with a risk of calcium, vitamin D, and iron deficiency and cannot prevent long‐term complications, such as progressive liver and renal failure. With the deepening understanding of the pathogenesis of GSDI and the development of gene therapy technology, there is great progress in the treatment of GSDI. Here, we review the underlying molecular genetics and the current clinical management strategies of GSDI patients with an emphasis on promising experimental gene therapies.

## 1 INTRODUCTION

Glycogen storage disease type I (GSDI) was first described by von Gierke in 1929, so it is also known as von Gierke disease. Glycogen storage disease type I is an inherited metabolic disorder caused by abnormalities of enzymes or proteins that are involved in glycogenolysis and gluconeogenesis.^1^ GSDI can be classified into four different subtypes based on molecular genetic variants: GSDIa, GSDIb, GSDIc, and GSDId. GSDIc and GSDId cases have been reported rarely, and GSDIa and GSDIb subtypes are the most clinically recognized.^2^ The probability of GSDI is estimated to be 1 in 100,000 live births, and GSDIa accounts for approximately 80%.^3^ GSDIa and GSDIb are autosomal recessive disorders caused by glucose‐6‐phosphatase (G6Pase‐a) and glucose‐6‐phosphate‐transporter (G6PT) deficiency, respectively.^4^ Both G6Pase‐a and G6PT are embedded in the membrane of the endoplasmic reticulum and are functionally coupled to maintain interprandial glucose homeostasis. Functionally, G6PT transports glucose‐6‐phosphate (G6P) from the cytoplasm to the endoplasmic reticulum lumen, where G6Pase‐a hydrolyzes G6P into glucose and inorganic phosphate.^5^

The primary metabolic abnormality in GSDIa and GSDIb is fasting hypoglycemia and lactic acidosis in the neonatal period, as G6Pase‐α and G6PT deficiencies result in the inability to hydrolyze G6P into glucose and phosphate. However, most newborns are free of symptoms as soon as they are fed foods containing enough glucose to prevent hypoglycemia, which usually only occurs at increased intervals between feedings. More commonly, the first symptom of GSDI is a protruding abdomen at about 3–6 months of age due to marked hepatomegaly, followed by progressive enlargement of the liver. Clinical features include doll‐like facies, growth retardation, short stature, and a distended abdomen owing to apparent hepatomegaly and nephromegaly. The high G6P level is shunted to alternative metabolic pathways, especially de novo lipogenesis, causing abnormal biochemical manifestations, including hypoglycemia, hyperlipidemia, hyperuricemia, hyperlactatemia, and hypertriglyceridemia.^5^, ^6^ In most of the patients with GSDI, liver enlargement decreases with age; however, 75% of patients older than 25 years are at risk for developing hepatocellular adenomas (HCA), of which 10% of HCA patients undergo malignant transformation into hepatocellular carcinoma (HCC).^7^ Moreover, patients with GSDI can also develop long‐term nephropathy due to excessive accumulation of glycogen and lipids in the kidney.^8^ Liver transplantation and kidney transplantation are the ultimate curative therapy for patients with GSDI.^9^ Neutropenia and myeloid dysfunction are unique to patients with GSDIb, which are associated with recurrent bacterial infections and enterocolitis.^10^ In addition to the presence of symptoms and a wide spectrum of characteristic biochemical abnormalities mentioned above, molecular genetic testing, and/or enzymatic testing of liver biopsy tissue provide clues to the diagnosis of GSDI. In patients with GSDI, the liver, kidney, and small intestinal mucosa are impaired due to excessive glycogen storage, and the cardiovascular system is typically unaffected; however, there were several early case reports showing pulmonary hypertension occurred in patients with GSDI.^11^, ^12^ Pulmonary hypertension is a very rarely seen complication, which has a very bad prognosis and its physiopathology is poorly understood.

Both GSDIa and GSDIb are fatal in childhood, if left untreated. The primary treatment for patients with GSDI focuses on dietary therapy, including continuous nocturnal nasogastric/intragastric infusion of glucose or oral administration of uncooked cornstarch to control glycemia and associated metabolic abnormalities.^4^, ^13^ Proper dietary management is helpful to prevent hypoglycemia and alleviate complications. Uncooked cornstarch (UCS) is an option starch choice to manage hypoglycemia for patients because of its slow‐release carbohydrate properties.^14^ However, enzyme replacement therapy is not appropriate for patients with GSDI because both G6Pase‐α and G6PT are highly hydrophobic transmembrane proteins. Gene therapy offers therapeutic advantages for diseases caused by defective and abnormal genes, especially monogenic diseases.^15^ Gene therapy involves the use of vectors to deliver the target gene to the relevant tissues or organs. There are two gene therapy strategies according to different delivery systems: viral vector‐based delivery and non‐viral vector‐based delivery. The viral vector‐based delivery includes lentiviral vectors (LV), retroviral vectors, adenoviral (Ad) vectors, adeno‐associated virus (AAV) vectors, and the like. The non‐viral vector‐based delivery can include cationic polymer vectors, liposome vectors, and nanoparticle vectors.^16^ There are numerous preclinical trials to investigate the viral vectors, which have been reported to account for 70% of gene therapy programs.^17^ An effective viral vector should be capable of delivering the transgene stably and efficiently to the tissue of interest. AAV is the most promising gene transfer vector for the treatment of glycogen storage disease type I due to its excellent safety profile, low immunogenicity, and durable and efficient transgene expression.^18^ The AAV vectors encoding different species G6Pase/SLC37A4 directed by the specific promoter/enhancer are administered to GSDI dogs or mice models that closely mimic severe GSDI in humans.

In this review, we will introduce the current understanding of the molecular genetics underlying GSDIa and GSDIb. We then summarize the clinical manifestations and pathologic phenotypes of GSDI on hepatic and renal metabolism as well as immune system. Lastly, we overview the current therapeutic strategies for GSDI including: (1) continuous dietary/nutritional therapies for GSDI patients, (2) conventional drug treatment for GSDI patients, and (3) viral vector‐mediated gene delivery with an emphasis on AAV‐based gene delivery systems.

## 2 MOLECULAR GENETICS OF GLYCOGEN STORAGE DISEASE TYPE I DISORDERS

### 2.1 Molecular pathogenesis of glycogen storage disease type Ia

**FIGURE 1:**
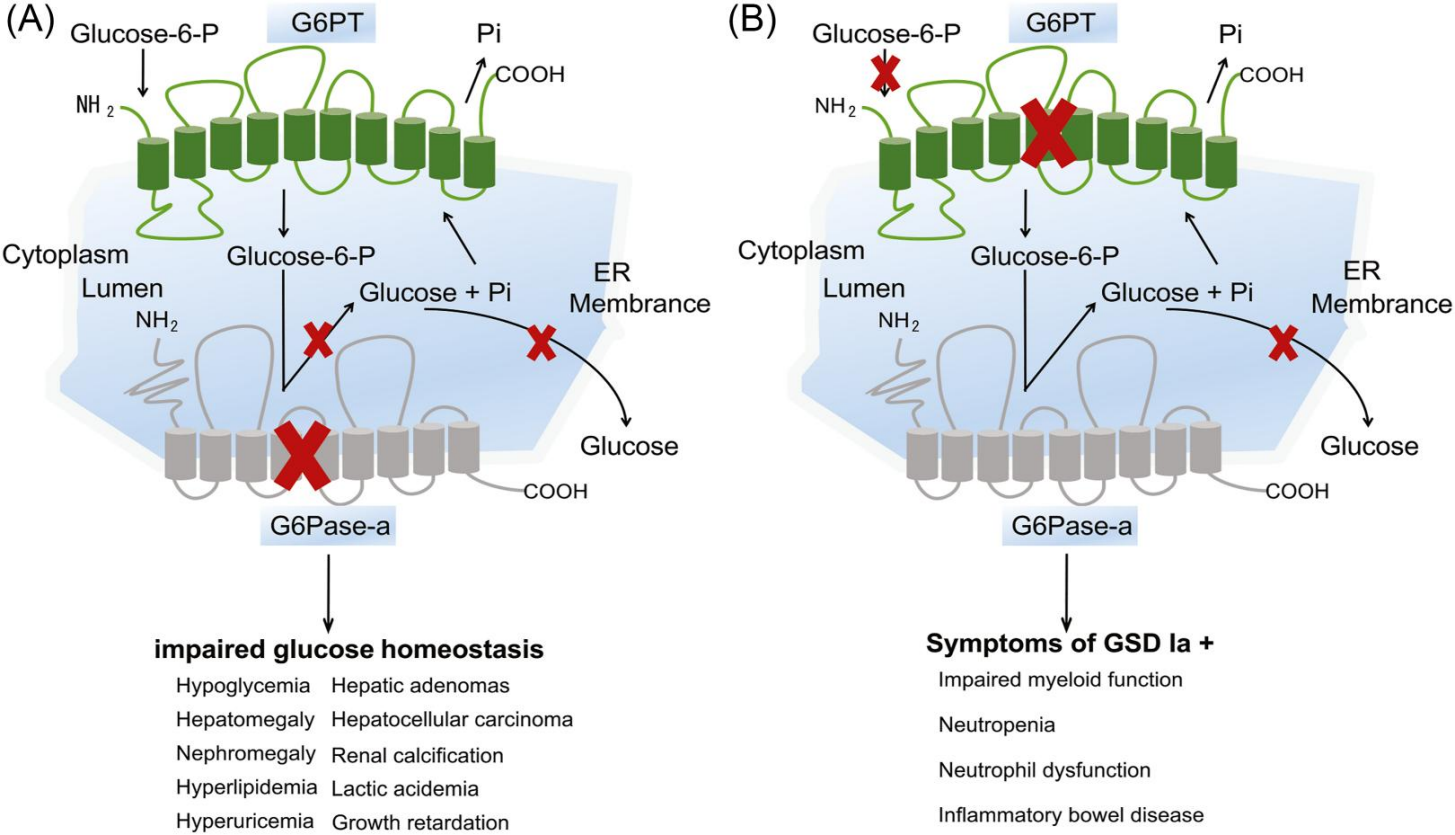
Potential targets of disrupted glucose homeostasis. Glucose‐6‐phosphatase‐α (G6Pase‐α) and glucose‐6‐phosphate transporter (G6PT) are shown embedded within the membrane of the endoplasmic reticulum (ER). In gluconeogenic organs, such as liver, kidney, and intestine, G6PT couples with G6Pase‐α to maintain interprandial blood glucose homeostasis. G6PT transports glucose‐6‐phosphate (G6P) from the cytoplasm to the lumen of the ER, where G6Pase‐α hydrolyzes G6P into glucose and inorganic phosphate. (A) Glycogen storage disease type Ia (GSDIa) is a glycogen metabolic disorder and manifests hypoglycemia and secondary metabolic symptoms. (B) Glycogen storage disease type Ib (GSDIb) is a metabolic and immune disorder. GSDIb manifests overlapping and distinct phenotypes compared with GSDIa.

Glycogen storage disease type Ia (GSDIa; OMIM#232200) is an autosomal recessive disorder that causes abnormal carbohydrate metabolism due to deleterious mutations in the glucose‐6‐phosphatase (G6Pase‐a or *G6PC*, OMIM*613742).^19^, ^20^ Human *G6PC* gene is a single‐copy gene composed of five exons, localized on 17q21.31, encoding a highly hydrophobic, 357 amino‐acid glycoprotein.^21^ To date, 118 *G6PC* mutation types have been identified in GSDIa patients, including 89 missense/nonsense, 5 splicing, 1 regulatory, 19 small insertions/deletions, 3 indels, and 1 gross deletions (via The Human Gene Mutation Database http://www.hgmd.cf.ac.uk/ac/gene.php?gene=G6PC). G6Pase‐α is expressed primarily in liver, kidney, and intestine and contributes to maintaining interprandial blood glucose homeostasis by reversibly hydrolyzing G6P into glucose and phosphate in the final common reaction of glycogenolysis and gluconeogenesis.^21^ In gluconeogenic organs, G6Pase is embedded in the membrane of the endoplasmic reticulum (ER) by nine transmembrane helices and has the amino‐terminus (NH2) located in the lumen, while the carboxyl‐terminus located in the cytoplasm^22^ (Figure 1A). Based on the crystal structure of the vanadium haloperoxidase, the active site residues of G6Pase‐a are all located on the lumen side of endoplasmic reticulum (ER) comprising Lys‐76, Arg‐83, His‐119, Arg‐170, and His‐176.^22^, ^23^, ^24^ The current paradigm for the G6Pase‐α reaction mechanism is that His‐176 acts as a nucleophile that covalently bound the phosphate of G6P forming a phosphohistidine‐enzyme intermediate.^22^

### 2.2 Molecular pathogenesis of glycogen storage disease type Ib

Glycogen storage disease type Ib (GSDIb, OMIM#232220) is an autosomal recessive disorder caused by a defect in the glucose‐6‐phosphate transporter (G6PT1) encoded by the solute carrier family 37 members 4 (*SLC37A4*) gene.^19^ Human *SLC37A4* gene is a single‐copy gene composed of nine exons, localized on 11q23.3. G6PT encodes 429 amino‐acid protein consisting of 10 transmembrane helices on the endoplasmic reticulum membrane with their amino‐terminus and carboxyl‐terminus located in the cytoplasm.^25^, ^26^ To date, 115 *SLC37A4* gene mutation types have been identified, including 65 missense/nonsense, 18 splicing, 1 regulatory, 27 small insertions/deletions, 2 indels, and 2 gross deletions (via The Human Gene Mutation Database, www.hgmd.cf.ac.uk/ac/gene.php?gene=SLC37A4). G6PT is ubiquitously expressed in all tissues and acts as a phosphate‐linked transporter, transporting the translocation of cytoplasmic G6P into the lumen of the endoplasmic reticulum, exchanging inorganic phosphate stored in the lumen.^26^ In the endoplasmic reticulum membrane, G6Pase‐α and G6PT are functionally coupled, and G6PT facilitates G6Pase‐α to catalyze the hydrolysis of the intracellular G6P into glucose to maintain interprandial glucose homeostasis (Figure 1B). Table 1 summarizes the similarities and differences between GSDIa and GSDIb characteristics.

**TABLE 1:** Similarities and differences of Glycogen storage disease type Ia (GSDIa) versus Glycogen storage disease type Ib (GSDIb).

Characteristics	GSDIa (von Gierke disease)	GSDIb
Affected gene	*G6PC*	*SLC37A4*
Enzyme deficiency (protein)	Glucose‐6‐phosphatase (G6Pase‐α)	Glucose‐6‐phosphate‐transporter (G6PT)
Chromosome location	17 (17q21.31)	11 (11q23.3)
Genome components	Five exons	Nine exons
Inheritance	AR	AR
Probability	80%	20%
Located	Liver, Kidney, Small intestine	Similarities with GSDIa + Neutrophils
Clinical description	Doll‐like facies, Growth retardation, Hepatomegaly, Nephromegaly, Hypoglycaemia, Hyperlipidemia, Hyperlactacidemia, Hyperuricemia, Hypertriglyceridemia	Symptoms of GSDIa + Neutropenia, Myeloid dysfunction
Long‐term complications	HCA HCC Renal disease, Pulmonary hypertension	Complications of GSDIa + recurrent bacterial infections, Inflammatory bowel disease

Abbreviations: AR, Autosomal recessive; HCA, Hepatocellular adenomas; HCC, Hepatocellular carcinoma.

## 3 CLINICAL MANIFESTATIONS AND PATHOLOGIC PHENOTYPES OF GLYCOGEN STORAGE DISEASE TYPE I DISORDERS

### 3.1 Liver phenotypes of Glycogen storage disease type I disorders

**FIGURE 2:**
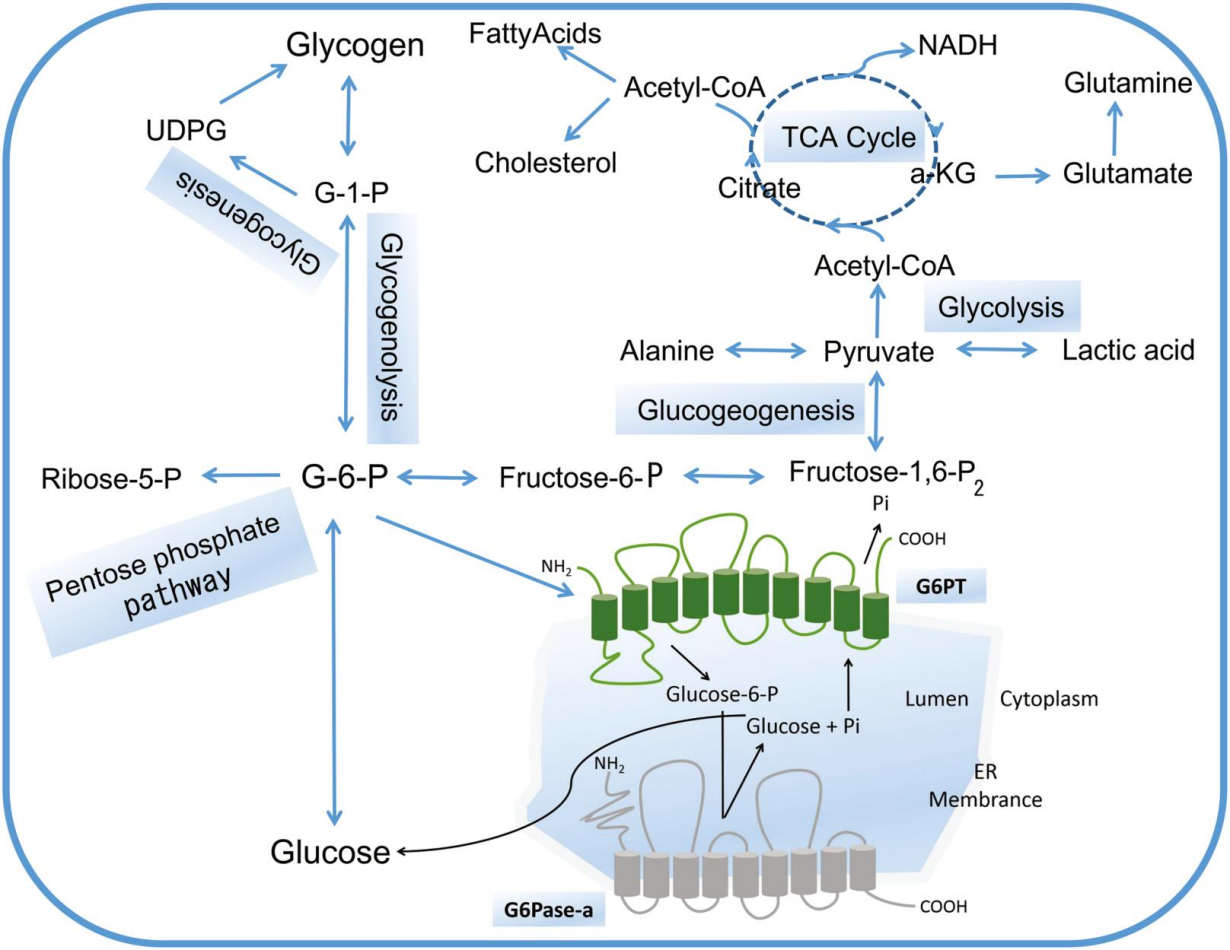
The essential role of G6P metabolism in gluconeogenesis. G6P can be hydrolyzed into glucose and inorganic phosphate in the terminal rate‐limiting step of gluconeogensis and glycogenolysis by G6Pase‐α. They are responsible for maintaining glucose homeostasis. Furthermore, G6P can be hydrolyzed to produce energy through glycolysis and TCA cycle. G6P can also provide ribose 5‐phosphate via the pentose phosphate pathway for nucleotide and nucleic acid biosynthesis. Conversely, G6P can be synthesized into glycogen via glycogenesis for energy storage.

The liver is a pivotal organ for regulating glucose homeostasis, keeping the blood glucose concentration at a relatively constant level, which is extremely important to ensure the utilization of various tissues and organs. Glucose is metabolized through a variety of direct competing pathways, glycolysis, tricarboxylic acid (TCA) cycle, pentose phosphate pathway (PPP), glycogenesis, glycogenolysis, gluconeogenesis, and other hexose metabolism^25^ (Figure 2). Glycogen storage disease type I is a genetic metabolic disorder in which G6Pase‐a or G6PT deficiency leads to extensive glycogen deposition. Both GSDIa and GSDIb are characterized by the aberrant utilization of glycogen, resulting in excessive glycogen accumulation in the cytoplasm of gluconeogenic organs (liver, kidney, and small intestine). Consequently, glucose production is insufficient and excessive G6P is shunted to alternative metabolic pathways, resulting in hypoglycemia and typical secondary metabolic abnormalities (e.g., hyperlactatemia, hypertriglyceridemia, hyperlipidemia, and hyperuricemia).^2^, ^27^ These findings are consistent with an observational study among 14 patients (11 GSDIa and 3 GSDIb), in which their plasma metabolomic profiles revealed alterations in metabolic flux mainly involving fuel and energy metabolism, lipids and fatty acids metabolism, amino acid and methyl‐group metabolism, urea cycle, and purine/pyrimidine metabolism compared to a cohort of age‐matched healthy controls.^28^

Excessive fat and glycogen accumulations in the liver and kidney contribute to progressive hepatomegaly and nephromegaly, respectively.^20^ Hepatomegaly tends to be prevalent in the younger children, resulting in abdominal protrusion; however, the size of the liver gradually decreases with age. Thus, dysregulated hepatic glycogen metabolic disorder can further trigger the development and/or progression of HCA, HCC, and eventually liver failure.^29^ HCA and HCC are severe long‐term complications in patients with GSDI.^30^ The severity of biochemical abnormalities and long‐term complications are correlated with the extent of hepatic G6PC activity deficiency in preclinical in vivo models. Several studies support this hypothesis: In a 70–90 weeks study, Lee al. showed that rAAV‐G6PC‐mediated gene therapy expressed 3%–63% of wild‐type hepatic G6Pase‐a activity in *G6pc* ^*−/−*^ mice, maintaining glucose hemostasis and preventing chronic HCA formation.^7^, ^31^ After that, they further showed that the rAAV‐vector‐treated *G6pc* ^*−/−*^ mice expressing 0.2% of normal hepatic G6Pase‐α activity experienced hypoglycemia, while expressing 0.5%–1.3% of normal hepatic G6Pase‐α activity showed no evidence of HCA formation. The authors concluded that 0.5%–1.3% of normal hepatic G6Pase‐α activity is the threshold of hepatic G6Pase‐α activity required to block HCA formation in GSDIa mice.^32^ However, Kwon al. showed *G6pt* ^*−/−*^ mice expressing <6% of normal hepatic G6PT activity developed HCAs after treating with rAAV‐GPE‐G6PT vectors.^33^ More recently, Rutten al. Demonstrated that affected mice with residual G6PC activity <60% showed a markedly increase in hepatomegaly, fasting hypoglycemia, hyperlactatemia, hypertriglyceridemia, glycogen contents, as well as oleate synthesis. These symptoms were more prominent in affected mice with G6PC activity <25%.^34^

### 3.2 Kidney phenotypes of Glycogen storage disease type I disorders

Kidney is also recognized as a major affected organ in patients with GSDI.^35^ The high level of G6P results in the activation of glycogenesis and de novo lipogenesis metabolic pathway, which is responsible for accumulation of renal glycogen and lipid. This phenomenon leads to a progressive decline in kidney function in patients with GSDI, most of whom are at risk for developing chronic kidney disease (CKD).^36^ One study showed that 14 patients with GSDI had a significant increase in glomerular filtration rate (GFR).^37^ This characteristic abnormality of glomerular hyperfiltration is firstly identified in patients with GSDI, followed by microalbuminuria, hypercalciuria, hypocitraturia, and later proteinuria.^8^, ^38^ About 70% of patients involved progress to proteinuria, and probably 30% develop end‐stage renal disease demanding dialysis. In some patients, renal function has deteriorated and gradually developed to nephrolithiasis, nephrocalcinosis, and eventually renal failure.^39^ The underlying mechanism of kidney disease in patients with GSDI and its molecular pathways involved needs to be further elucidated.

A mouse model of kidney‐specific G6Pase deficiency (K. *G6pc* ^*−/−*^ mice) was generated to characterize the development of nephropathy in GSDIa by Clar et al.^40^ The study showed that renal G6PC knockout led to excessive glycogen accumulation in proximal tubules, resulting in tubular dilation and nephromegaly, as well as microalbuminuria after 6 months of G6pc deletion.^40^ Several studies have shown that the activation of renin‐angiotensin system (RAS), enhance of renal oxidative stress, increased expression of transforming growth factors, and insufficient energy in renal tubular epithelial cells are associated with glomerular damage and nephropathy in the GSDI mice.^6^, ^41^, ^42^

### 3.3 Immune dysfunction in Glycogen storage disease type I disorders

Both GSDIa and GSDIb patients present generally similar clinical symptoms, whereas GSDIb patients have an impact in immune disorders, including neutropenia and myeloid dysfunction, causing recurrent bacterial infections, inflammatory bowel disease, and mucosal lesions.^1^, ^33^, ^43^, ^44^ The therapeutic approach for patients with GSDIb is the administration of granulocyte‐colony stimulating factor (G‐CSF) in order to increase the number of neutrophils and mitigate the incidence of infections.^25^ A study investigated the hypothalamus‐pituitary‐thyroid axis in patients with GSDI demonstrated that 57% patients with GSDIb were observed to have an increased prevalence of thyroid autoimmunity and hypothyroidism.^45^ The underlying mechanism between neutropenia and/or neutrophil dysfunction in GSDIb and its relationship to G6P homeostasis is unclear.

In 2003, Kuijpers et al. observed a significantly higher level of apoptosis in circulating neutrophils in patients with GSDIb, which may be associated with granulocyte dysfunction.^46^ A mass spectrometric glycomic profiling of neutrophils in GSDIb suggested a novel explanation that hypoglycosylation of the electron transporting subunit of NADPH oxidase, gp91phox, was responsible for the correlation of neutrophil dysfunction and neutropenia in GSDIb patients.^47^ Most recently, Veiga‐da‐Cunhaet al. found 1,5‐anhydroglucitol‐6‐phosphate (1,5‐AG6P), a structural analog of G6P, accumulated in patients with G6PT or G6PC3 deficiency.^48^ In patients with GSDIb, 1,5‐AG6P cannot be transported from the cytosol into the ER by G6PT, resulting in the excessive accumulation of 1,5‐AG6P in neutrophils.^44^ The exact relationship of 1,5‐AG6P accumulation and G6PT mutations was confirmed by Veiga‐da‐Cunhaet al. As they found that cytosolic accumulating noncanonical metabolite 1,5‐AG6P leads to neutropenia and neutrophil dysfunction in G6PT‐deficient patients.^48^ It is well known that 1,5‐AG6P is an inhibitor of hexokinases, and mature neutrophils primarily depend on glycolysis for energy. The improvement in neutrophil function is mainly due to the decrease of intracellular 1,5‐AG6P concentration.

## 4 CLINICAL MANAGEMENT AND THERAPIES OF GLYCOGEN STORAGE DISEASE TYPE I DISORDERS

### 4.1 Dietary/nutritional therapies for Glycogen storage disease type I disorders

Before gene therapy technology had been developed, most of the early investigations focused on using dietary management and/or conventional drugs to treat GSDI. Currently, there are no targeted pharmaceutical interventions for patients with GSDI. Regimented dietary therapy is taken to treat this metabolic disruption disease. In the 1960s, portacaval shunts were used to correct patients with congenital metabolic errors. This surgical strategy shunts blood from the portal venous system into the vena cava system, which is an excellent way to favorably prevent dietary carbohydrates from passing through the liver and allow glucose more readily available to peripheral tissues.^13^, ^49^ Following this procedure, portacaval shunts coincidentally relieve hypoglycemia and avoid glycogen deposition in the liver; however, hypohepatia, jaundice, ascites, and even hepatic encephalopathy will occur after portacaval shunting.

In the 1970s, continuous nutrition therapy, including total parenteral nutrition (TPN), nocturnal nasogastric infusion, and nighttime intragastric feeding, was introduced to well prevent universally fatal hypoglycemia.^13^ These clinic trials demonstrated that patients with type 1 disease receiving intragastric feeding with a high‐glucose formula combined with frequent daytime feeding can prevent hypoglycemia and reverse metabolic anomalies associated with GSDI^50^; however, continuous feeding is possibly interrupted especially during the night. Hence, parents of affected infants must take some measures to avoid interruption in nighttime feeding because recurrent hypoglycemia can lead to seizures and even death from pump failures.^6^ The rate administration of carbohydrates is not a constant formula, and plasma glucose concentration needs to be considered to minimize organic acidemia in children with GSDI. The carbohydrate infusions rates are calculated based on the body weight and age: the rate of administration for children under 6 years is 7–9 mg per kg per minute, for older children such as schoolchildren or adolescents is 5–6 mg per kg per minute for 10 h, and for adults is 3–4 mg per kg per minute for 8–10 hours.^51^, ^52^, ^53^ Interestedly, the study's mathematical formula showed that estimated glucose requirements were linearly correlated with brain size, rather than to weight and age.^13^

Subsequent, investigations tested numerous starches with the intention to find a slow‐release carbohydrate source. In particular, uncooked cornstarch (UCS) is an ideal starch to manage hypoglycemia for children and adults with GSDs.^14^ Compared to different starchy foods, cornstarch has several characteristics in preventing hypoglycemia. First, cornstarch digests and absorbs slowly, which can avoid a rapid increase in blood glucose in a short period of time and maintain glucose concentrations for a longer time. Consequently, cornstarch results in lower insulin concentration, which is beneficial because high insulin concentration increases the risk of hypoglycemia and decreases the production of lactate and ketone bodies.^13^, ^14^, ^51^ A smaller amount of cornstarch was used to maintain normal glucose concentrations, and the recommended dosage for children is 1.75–2.5 g/kg every 6 h.^51^ Over the past 50 years, strict dietary therapies have transformed GSDs from a fatal metabolic disorder to one with a good prognosis, extending the lifespan and improving the quality of life of GSDs patients.^13^

### 4.2 Adjunct drug therapies for Glycogen storage disease type I disorders

Adjunct drug therapies have improved GSDI patient's management (Table 2). Clinically, renin‐angiotensin system (RAS) blockers, such as angiotensin‐converting inhibitors (ACEi) or angiotensin receptor blockers (ARBs), have been reported to effectively reverse hypertension and delay progression of chronic renal failure in patients with GSDI.^38^, ^54^, ^55^, ^56^ Patients with GSDIb are often treated with G‐CSF to improve symptoms of inflammatory bowel disease (IBD) and prevent severe neutropenia.^57^, ^58^, ^59^ A study reviewed medical records of 103 patients with GSDIb subjected to drug therapy with G‐CSF, and data indicated that G‐CSF can stimulate peripheral blood neutrophil counts and ameliorate the occurrence of infections.^60^ Other studies have shown that vitamin E is equally useful for alleviating disease manifestation associated with neutropenia.^61^, ^62^ However, there has been increasing evidence that long‐term G‐CSF administration can lead to the occurrence of malignancies (e.g., MDS or AML).^57^, ^63^ Thus, more effective and safe medications are required to treat patients with GSDIb. Empagliflozin, an inhibitor of the renal glucose cotransporter SGLT2, has emerged as a new and appropriate treatment option for neutropenia and neutrophil dysfunction in patients with GSDIb.^64^ A report of the clinical data from 112 pediatric and adult individuals with GSDIb clearly showed that empagliflozin markedly improves neutrophil counts and has a positive effect for neutrophil dysfunction‐related symptoms.^65^, ^66^ Empagliflozin has also been used to treat GSDIb patients, resulting in decreased serum 1,5‐AG and neutrophil 1,5‐AG6P levels.^43^, ^44^, ^67^, ^68^

**TABLE 2:** Current therapeutics used in Glycogen storage disease type I (GSDI) models or GSD patients.

Agents	Targets	Subject	Results	References
Bezafibrate	PPAR	*G6pc−/−* mice	Decreased hepatic glycogen and triglyceride partially reversed hepatic autophagy defect Increased AAV vector‐mediated genome editing	71
		GSD‐Ia dog		
Fenofibrate	PPAR‐α	*L.G6pc −/−* mice	Decreased hepatic glycogen and triglyceride and renal triglyceride Prevented liver or kidney damages	70, 72, 73
		*K.G6pc −/−* mice		
		*G6pc −/−* mice		
VK2809	THR *β*	*G6pc −/−* mice	Decreased hepatic triglyceride Stimulated hepatic autophagic flux Promote mitochondrial β‐oxidation	69
G‐CSF	Neutrophils	GSDIb patients	Raised blood neutrophil counts Ameliorates inflammatory bowel symptoms Increased risk of myelodysplastic syndrome (MDS) or acute myeloid leukemia (AML)	57, 58, 59, 60, 63, 63
Empagliflozin	SGLT2	GSDIb patients	Reduced plasma 1,5AG levels Reduced intracellular 1,5AG6P Improved bowel health, growth, and laboratory parameters	43, 44, 64, 65, 66, 67, 68
ACEi	ACE	GSDI patients	Decreased glomerular filtration rate Delayed progression of chronic renal failure	37, 54, 55, 56
Vitamin E	Antioxidant	GSDIb patients	Raised neutrophil counts Reduced frequency and severity of infections	61, 62

Abbreviations: ACEi, angiotension converting enzyme inhibitors; G6pc, glucose 6 phosphatase‐α; G6pt, glucose 6 phosphate transporter; G‐CSF, granulocyte‐colony stimulating factor; GSDI, glycogen storage disease type I; K.G6pc −/− mice, Kidney‐specific G6pc deficient mice; L.G6pc −/− mice, Liver‐specific G6pc deficient mice; PPAR, peroxisome proliferator‐activated receptor; PPAR‐α, peroxisome proliferator‐activated receptor‐alpha; SGLT2, sodium‐dependent glucose transporters 2; THR β, thyroid hormone receptor β.

In addition, there are several novel pharmacological agents developed for the treatment of GSDIa patients. VK2809, a thyroid hormone receptor agonist selective for liver tissues, was used to treat GSDIa mice. VK2809 stimulation decreased hepatic triglyceride levels by inducing autophagy and mitochondrial biogenesis promoting fatty acid *β*‐oxidation.^69^ Similarly, fenofibrate and bezafibrate are used to improve hepatic and/or renal lipid metabolism and reduce glycogen storage in neonatal *G6pc* ^*−/−*^ mice through the induction of autophagy to promote *β*‐oxidation of fatty acids.^70^, ^71^ Furthermore, fenofibrate, via the promotion of fatty acid *β*‐oxidation, significantly decreased the hepatic and renal glycogen and triglyceride accumulations, and prevented liver injury and nephropathy in L.*G6pc* ^*−/−*^ and K.*G6pc* ^*−/−*^ mice.^72^ These findings suggested that the reduction of hepatic lipid by pharmacologically induction autophagy may be a novel therapeutic strategy for GSDIa.^73^

### 4.3 Gene therapies for Glycogen storage disease type I disorders

#### 4.3.1 Adenovirus vector‐mediated gene therapies for Glycogen storage disease type I disorders

Somatic gene therapy is a promising treatment for inherited diseases caused by mutations in genes, such as GSDIa and GSDIb^18^ (Table 3). Gene therapy studies have demonstrated the first and early generation adenovirus vectors expressing G6Pase‐a or G6PT effectively normalized the metabolic abnormalities in GSDI mice and canine models.^26^ In 2000, Zingone al. infused an adenovirus vector carrying murine G6Pase gene into *G6pc* ^*−/−*^ mice (Ad‐mG6Pase) via the retro‐orbital vein and restored hepatic G6Pase activity to 19% of that of *G6Pase* ^*+/+*^ mice, thereby improving pathological manifestations and increasing the survival rate after weaning to 100%. However, the kidneys of adenovirus‐infused *G6pc* ^*−/−*^ mice showed little or no detectable expression of the G6Pase.^74^, ^75^ To compensate for the limited efficiency of adenovirus alone, Sun al. proposed a strategy of co‐administration with adenovirus and adeno‐associated virus type 2 vectors (Ad/AAV‐mG6Pase) to maintain sustained G6Pase expression in the liver and kidney of neonatal *G6pc* ^*−/−*^ mice.^76^ In addition, neonatal *G6pt* ^*−/−*^ mice were infused intravenously with adenoviral vector containing human G6PT (Ad‐hG6PT) effectively delivered G6pt mRNA to the liver, bone marrow, and spleen and corrected neutropenia as well as myeloid abnormalities.^5^ Nevertheless, it is known that Ad‐mediated transient transgene expression is not suitable for maintaining long‐term G6Pase activity and can cause serious immunogenicity. To achieve sustained transgene expression and alleviate viral toxicity, a helper‐dependent adenovirus vector encoding G6Pase (HDAd‐G6Pase) was administered to the GSDIa murine^77^ and canine model,^78^ directed by the human apolipoprotein AI promoter/enhancer. As a result, HDAd‐mediated delivery of G6Pase effectively corrected physiological and biochemical abnormalities in G6Pase‐KO mice and GSDIa dogs, markedly prolonging median survival time to 7 months compared to 5–6 weeks in Ad‐treated mice.^76^, ^77^

**TABLE 3:** List of experimental gene therapies in Glycogen storage disease type I (GSDI) models.

Author/year	Gene	Treatment vector	Promoter enhancer	Species models	Infusion‐method‐age	Results
Zingone et al. 2000	Murine G6PC	Adenoviral	RSV	G6pc −/− mice	Retro‐orbital vein (14‐day‐old)	Restored hepatic G6Pase activity to 19% of G6Pase+/+
Beaty et al. 2002	Canine G6PC	AAV	Mouse albumin promoter/enhancer	GSD Ia dogs	Intravenous (3‐ or 4‐day‐old)	Resulted in sustained hepatic G6Pase activity; improved liver histology and biochemical parameters
Mao‐Sen et al. 2002	Murine G6PC	Co‐infused adenoviral; AAV	RSV; CBA promoter/CMV enhancer	G6pc −/− mice	Temporal vein (1‐day‐old); intravenous (2‐week‐old)	Resulted in sustained G6Pase expression in both the liver and the kidney; corrected the murine GSD‐Ia disease
Koeberl et al. 2006	Canine G6PC	AAV8	Nucleotides −1372 to −11 of the canine G6PC 5′‐fanking sequence	G6pc −/− mice	Intravenous (2‐week‐old)	Prolonged median survival to 7 months; restored hepatic G6Pase activity to 25% of normal level at 7 mouths of age
Ghosh et al. 2006	Murine G6PC	AAV1; AAV8	CBA promoter/CMV enhancer	G6pc −/− mice	Temporal vein (1‐ or 2‐day‐old); retro‐orbital vein (1‐week‐old)	Increased hepatic G6Pase activity; improved survival; corrected the metabolic abnormalities for the 57 weeks
Koeberl et al. 2007	Canine G6PC	HDAd	Human apolipoprotein AI	G6pc −/− mice	Intravenous (2‐week‐old)	Increased hepatic G6Pase activity over tenfold between 3 days and 28 weeks; prolonged median survival to 7 months; reduced liver glycogen storage; reversed the physiological and biochemical abnormalities
Yiu et al. 2007	Human SLC37A4	Adenoviral	CMV	G6pt −/− mice	Temporal vein (1 or 2‐day‐old); retro‐orbital vein (2‐week‐old)	Restored G6PT mRNA level in the liver, bone marrow, and spleen; corrected metabolic and myeloid abnormalities
Koeberl et al. 2008	Human G6PC	AAV2/8	Nucleotides −298 to +128 of the human G6PC 5′‐fanking sequence	G6pc −/− mice; GSD Ia dogs	Retro‐orbital sinus (12 ± 1 day of age mice); Venipuncture of the jugular vein (3 days of age dogs)	Prevented hypoglycemia, corrected liver G6Pase deficiency; reduced liver glycogen storage in GSD‐Ia mice and dogs; prolonged survival
Yiu et al. 2009	Human SLC37A4	AAV8	CBA promoter/CMV enhancer	G6pt −/− mice	Temporal vein (1‐day‐old)	Hepatic G6PT activity was 50% of wild‐type at 2 weeks post‐infusion but declined to 3% of wild‐type levels by age 6–72 weeks
Grinshpun et al. 2010	Human G6PC	FIV	EF‐1α, CMV, or hAAT	G6pc −/− mice	Temporal vein (1‐day‐old); retro‐orbital sinus (7 days of age)	Normalized blood glucose levels; improved body weight, and decreased accumulation of liver glycogen
Yiu et al. 2010	Human G6PC	AAV8	Nucleotides −2864 to −1 of the human G6PC 5′‐fanking sequence	G6pc −/− mice	Temporal vein (2‐day‐old); retro‐orbital sinus (2‐ or 4‐week‐old)	Normalized blood glucose levels, blood metabolites, hepatic glycogen, and hepatic fat
Weinstein et al. 2010	Murine G6PC	rAAV2/8; rAAV2/1	CBA promoter	GSD Ia dogs	Jugular vein (1 day old); portal vein injection (20‐week‐old)	Corrected of the GSD Ia phenotype
Luo et al. 2011	Human G6PC	AAV2/7	Human G6PC 5′‐fanking sequence	G6pc −/− mice	Retro‐orbital sinus (12 ± 1 day‐old)	Prevented hypoglycemia; reduced glycogen content in the liver and renal; reduced Albuminuria and renal fibrosis
Crane et al. 2011	Human G6PC	HDAd	Human apolipoprotein AI	GSD Ia dogs	Jugular venipuncture (3‐day‐old dogs)	Normalized blood glucose levels between 6 and 22 months elevated hepatic G6Pase activity and reduced glycogen content; prolonged survival
Demaster et al. 2012	Human G6PC	AAV2/7; AAV2/8; AAV2/9	Data not available	GSD Ia dogs	Jugular venipuncture (2‐ or 3‐day‐old)	Normalized glycogen content; restored hepatic G6Pase activity to 40% of normal in female dogs at 7 mouths of age
Lee et al. 2012	Human G6PC	AAV2/8	Human G6PC 5′‐fanking sequence	G6pc −/− mice	Retro‐orbital sinus (2‐ or 4‐week‐old)	Prevented chronic HCA formation; restored hepatic G6Pase activity more than 3%–100% of wild‐type
Brooks et al. 2013	Human G6PC	AAV2/8; AAV2/9	Human G6PC 5′‐fanking sequence	G6pc −/− mice; GSD Ia dogs	(2‐week‐old) (1–3‐day‐old)	Improved growth; promoted long‐term survival
Lee et al. 2015	Human G6PC	AAV2/8	Human G6PC 5′‐fanking sequence	G6pc −/− mice	Retro‐orbital sinus (2‐week‐old)	Prevented HCA formation and hepatic steatosis; expressed 0.5%–1.3% of wild‐type hepatic G6Pase‐α activity grow normally to age 75–90 weeks
Kim et al. 2015	Human G6PC	AAV2/8	Human G6PC 5′‐fanking sequence	G6pc −/− mice	Retro‐orbital sinus (2‐week‐old)	Activated signaling by hepatic carbohydrate response element binding protein
Landau et al. 2016	Human G6PC	AVV2/8‐ZFN; AVV2/9‐ZFN; AVV2/8‐RoG6P; AVV2/9‐RoG6P	Thyroid hormone binding globulin liver‐specific promoter; human G6PC minimal promoter	G6pc −/− mice	Retro‐orbital (12 ± 1‐day‐old)	Improved survival; improved biochemical correction; improved vector persistence and efficacy, and lower mortality
Kwon et al. 2017	Human SLC37A4	rAAV	2.8 kb human G6PC; 1.62 kb human G6PT; 610‐bp kb human G6PT	G6pt−/− mice	Temporal vein (1‐day‐old); Retroorbital sinus (4‐week‐old)	Established the threshold of hepatic G6PT activity required to prevent tumor formation; protected against age‐related obesity and insulin resistance
Kim et al. 2017	Human G6PC; human co‐G6PC	rAAV	Human G6PC 5′‐fanking sequence	G6pc −/− mice	Retro‐orbital sinus (2‐week‐old)	rAAV‐co‐G6PC vector is more effective than the rAAV‐G6PC vector; establish the threshold of hepatic G6Pase‐α activity required to prevent HCA/HCC
Lee et al. 2018	Human G6PC	rAAV1	2864‐bp human G6PC	GSD Ia dogs	Jugular venipuncture (1‐day‐old); portal vein (2–6‐month‐old)	Improved survival; prevented focal hepatic lesions or renal abnormalities
Brooks et al. 2018	Human G6PC	AAV2/7; AAV2/8; AAV2/9	Human G6PC minimal promoter	GSD Ia dogs	Data not available	Developed renal failure; exhibited progressive kidney disease histologically
Kang et al. 2019	Human G6PC	AVV2/9‐RoG6P; AVV2/9‐ZFN	Human G6PC minimal promoter	Liver G6pc −/− mice	Intravenous (7–8weeks‐old)	Corrected hepatic G6Pase deficiency; suppressed HCA and HCC
Cho et al. 2019	Human G6PC	rAAV	Human G6PC 5′‐fanking sequence	L‐ G6pc −/− mice	Retroorbital sinus (at 53 weeks post G6pc gene deletion)	Restored hepatic G6Pase‐α expression; prevented de novo HCA/HCC development
Zhang et al. 2019	Human co‐G6PC‐S298 C	rAAV	Human G6PC 5′‐fanking sequence	G6pc −/− mice	Retro‐orbital sinus (2‐week‐old)	Efficacy of the rAAV‐hG6PC‐S298 C vector was3‐fold higher than that of the rAAV‐hG6PC‐WT vector
Zhang et al. 2020	Human G6PC‐S298 C	rAAV	Human G6PC 5′‐fanking sequence	G6pc −/− mice	Retro‐orbital sinus (2‐week‐old)	Treated with a lower dose to survive long term; expressing ≥3% of normal hepatic G6Pase‐a activity do not develop hepatic tumors;
Arnaoutova et al. 2021		rAAV8	U6 promoter	G6pc‐R83/R83 C mice	Remporal vein (newborn)	Displayed normalized blood metabolites expressing ≥3% of normal hepatic G6Pase‐a activity; increased 16‐week survival rate from 0% to 100%

Abbreviations: AAV, adeno‐associated virus; CBA, chicken β‐actin; CMV, cytomegalovirus; FIV, Feline immunodeficiency virus; G6PC, glucose 6 phosphatase‐α; G6PT, glucose 6 phosphate transporter; GSDI, glycogen, storage disease type I; RSV, rous sarcoma virus.

#### 4.3.2 Adeno‐associated virus vector‐mediated gene therapies for Glycogen storage disease type I disorders

In contrast to first‐generation adenovirus vector‐mediated gene therapy, recombinant adeno‐associated virus (rAAV) has several unique characteristics as a gene delivery vector. There is no direct evidence that rAAV can cause vector genome‐mediated host genotoxicity in humans, making rAAV become the primary vector for gene transfer in vivo.^18^ Investigators have identified 12 human AAV serotypes (AAV1 to AAV12), of which the most commonly used rAAV serotypes include AAV2, 5, 8, and 9. In 2002, Beaty et al. firstly developed an AAV vector (AAV‐AlbcG6PGH) containing mouse albumin promoter/enhancer driving the expression of canine G6Pase. The administration of this agent to GSDIa canines resulted in sustained G6Pase expression and improvement in hepatic histology and relevant biochemical parameters.^79^ Different serotypes of AAV exhibit different tropisms, which are mainly dependent on the interaction of the amino acid sequence and structure of AAV capsid with host cytokines, including cell surface receptors, signaling molecules, and co‐receptors.^80^ One study found that AAV serotype 8 (AAV8) has become one of the preferred vector serotypes for liver‐targeted gene therapy because it is a highly liver tropic and efficient vector with low preexisting immunity.^81^ Koeberl et al. used AAV serotype 8‐based vectors expressing G6Pase to administrate to *G6pc* ^*−/−*^ mice or GSDIa dogs and that showed they could prolong median survival and restore hepatic G6Pase activity in affected models.^39^, ^82^ In subsequent years, a large body of research has systemically shown that the administration of rAAV pseudotype 2/8 or 2/7 vectors expressing the wild‐type human G6Pase‐α (rAAV‐G6PC) achieve high efficiency of transduction and correct metabolic abnormalities in the infused *G6pc* ^*−/−*^ mice or GSDIa dogs, directed by the human G6PC promoter/enhancer (GPE).^7^, ^9^, ^31^, ^32^, ^39^, ^83^, ^84^, ^85^, ^86^ More recently, Kim al. found that the *G6pc* ^*−/−*^ mice treated with rAAV‐G6PC and rAAV‐co(codon‐optimized)‐G6PC expressed more than 3% of normal hepatic G6Pase‐α activity and prevented the development of age‐related obesity and insulin resistance.^31^, ^87^

These evolutionary approaches of single amino acid variant have been widely used to improve the efficacy and potency of rAAV‐mediated gene transfer and therapy for GSD and reduce the adaptive immune response to the rAAV.^88^, ^89^, ^90^ Initially, Kim et al designed a rAAV vector expressing a codon‐optimized (co) G6Pase‐α (rAAV‐co‐G6PC) and found that rAAV‐co‐G6PC mediated more efficient hepatic G6Pase‐α expression than rAAV‐G6PC.^87^ Later, Zhang et al. constructed a rAAV‐G6PC‐S298 C vector with a single amino acid variant in the native human G6PC sequence. This rAAV‐G6PC‐S298 C vector mediated a threefold higher expression of G6Pase‐α activity than the native rAAV‐G6PC vector in the short‐term, which simultaneously enhanced and prolonged survival of the young mice under low dose of the vector.^88^ An alternative vector enabled researchers to further enhance initial G6Pase‐α enzymatic activity by simultaneously combining codon‐optimization with the S298 C variant (designated as rAAV‐co‐S298 C).^90^ Similarly, Cao et al. showed that serine (S) to cysteine (C) substitution at position 298 (S298 C) had elevated G6Pase‐α activity among 20 G6Pase‐α protein variants, while combining protein variant S298 C and codon optimization can maximize enzymatic activity.^89^

rAAV vector genomes are predominantly persisted in the nucleus in an extrachromosomal form (circularized dsDNA). AAV genome can mediate long‐term gene expression, relying solely on cellular proteins to convert the single‐stranded genome into stable circular and concatemeric episomal forms.^91^ However, AAV vectors can transduce cells by integrating nonhomologous chromosomal locations, which have been developed for the treatment of GSDIa.^92^, ^93^ The mouse ROSA26 locus is a ‘‘safe harbor’’ and is frequently used as a safe site for gene targeting by homologous recombination (HR). Mouse ROSA26 locus is a preferred site for gene targeting as it can achieve stable and efficient transgene expression without affecting the expression and function of other nearby endogenous genes.^94^ The engineered zinc finger nucleases (ZFNs) have been proposed as an optimal means of precise genome modification, which can generate site‐specific DNA double‐strand breaks in target genomic sequences. Studies have demonstrated the feasibility of gene targeting to the mouse ROSA26 locus directed by engineered ZFNs.^94^, ^95^ Studies have developed dual AAV vector‐mediated genome editing in G6Pase knockout mice, one vector (AAV‐ZFN) containing a ZFNs generating DNA double‐strand breaks in the *ROSA26* gene and one vector (AAV‐G6Pase) containing G6PC donor transgene targeting *ROSA26* locus. ZFN‐mediated targeted gene therapy integrates G6Pase gene cassette into the murine *ROSA26* safe harbor locus, significantly prolonging and enhancing G6Pase expression in treated *G6pc* ^*−/−*^ mice.^93^, ^96^

## 5 DISCUSSION AND FUTURE DIRECTIONS

Glycogen storage disease type I is a rare inherited disease characterized by the overaccumulation of G6P, since G6Pase‐a and G6PT deficiency resulted in the failure to hydrolyze G6P into glucose and phosphate in the final step of glycogenolysis and gluconeogenesis.^97^ The patients with GSDI suffer from severe hypoglycemia and metabolic disorder associated complications. Currently, there are no specific drugs for this disease, and the focus is on preventing hypoglycemia and mitigating progression of related complications. Over the past 50 years, the care of patients with GSDI has improved following elaborate dietary regimens, including continuous nutrition therapy, restriction of non‐utilizable sugars, and frequent cornstarch feeding. Patients with GSDI currently receive dietary therapies that have enabled patients to improve hypoglycemia and remove early symptoms and signs of the disease. However, dietary therapies have many limitations with a risk of calcium, vitamin D, and iron deficiency and cannot prevent long‐term complications, such as progressive liver and renal failure.^98^

The high levels of G6P accumulate in the liver and are shunted to alternative metabolic pathways, especially glycogen synthesis and de novo lipogenesis. These findings suggested that reducing hepatic lipid may be a novel therapeutic strategy for patients with GSDI. Furthermore, studies have shown that glycogen metabolism has been linked to autophagy, such as Pompe disease and Lafora disease.^99^, ^100^ Activation of autophagy is critical for energy utilization and clearance of intracellular lipid, which can preferentially hydrolyze intrahepatic lipid droplets to promote mitochondrial fatty acids *β*‐oxidation.^101^ A number of previous studies have demonstrated the use of drugs to promote hepatic autophagy in the treatment of GSDIa mice, such as VK2809, fenofibrate as well as bezafibrate.^69^, ^71^, ^102^ Recently, mediator complex subunit 1 (MED1) has play an important role in regulating hepatic autophagy, *β*‐oxidation of fatty acids, and mitochondrial function.^103^ The effect of MED1 on glycogen storage disease has not been studied and it may be an alternative drug for glycogen storage disease.

ACEi is currently one of the most effectual drugs for the treatment of hypertension and glomerular diseases. However, ACEi is unable to improve microalbuminuria and proteinuria.^38^ It may be the reason that G6P is overaccumulated in early patients with GSDI resulting in activating protein kinase C and regulating renal angiotensinogen.^104^ New agents are being developed for the treatment of renal damage in patients with GSDI. Transforming growth factor beta cytokine (TGF‐β) has been widely recognized a central participant of chronic renal disease. TGF‐β is upregulated and mediates chronic renal disease by promoting fibroblast proliferation, epithelial‐to‐mesenchymal transition, production of tubular and fibroblast extracellular matrix proteins (ECM), and podocyte injury.^105^ Thus, inhibiting TGF‐β axis and its crosstalk pathways can be a promising therapeutic strategy for renal disease.^106^

Considering that both G6Pase‐a and G6PT are highly hydrophobic transmembrane proteins, enzyme‐replacement therapy is not suitable for patients with GSDI. Up to now, gene therapy studies using GSDIa and GSDIb models of mice or dogs have shown that AAV vectors encoding G6Pase‐α deliver transgene to the liver or kidney can be directed by the chicken *β*‐actin promoter/CMV enhancer,^76^, ^107^, ^108^ the mouse albumin promoter/enhancer,^79^ the canine G6PC promoter,^82^ or the human G6PC promoter.^7^, ^31^, ^32^, ^85^, ^88^, ^90^ Unfortunately, these animal models have severe hypoglycemia and a short life expectancy, which make them unsuitable for studying the long‐term complications of gene therapy on GSDI. These studies have well‐demonstrated that the numbers of vector genomes could decline over time following rapid division of hepatocyte cell in young GSDIa mice; thus, the rAAV‐mediated gene therapy cannot be durably and efficiently expressed in transgenic tissues.^88^, ^90^, ^93^, ^96^ One study found that the significant increase in hepatic CD8^+^ lymphocyte counts was observed in *G6pc* ^*−/−*^ mice infused with AAV‐CBA at age 2 weeks; in contrast, low levels of hepatic CD8+ lymphocyte counts were observed with AAV8‐GPE under the same conditions.^83^ The results suggest the rapid decline and low efficacy of transgene expression are associated with the CBA promoter/CMV enhancer and there may be an inflammatory immune response against the AAV‐CBA vector. Previous gene therapy studies have used two different vectors, rAAV8‐GPE and rAAV8‐miGPE, encoding two human G6Pase‐α to treat the mouse model of GSDIa, directed by 2864‐bp of the human G6PC promote/enhancer and 328‐bp of minimal G6PC promote/enhancer, respectively. However, the results showed that the rAAV8‐GPE was more effective for hepatic G6Pase‐α expression than the rAAV8‐miGPE vector.^109^ The molecular mechanisms of promoters affecting vector genomes decline and transgene expression efficacy remain to be explored.

As one of the most promising gene therapy vectors, AAV vectors cannot be integrated into the host‐cell genomes and predominantly act as an episome in the host nucleus.^110^ However, AAV‐mediated gene therapy has some limitations, including (1) immune response to the viral vector due to preexisting neutralizing antibodies in the body, (2) transgene expression in progressively lost over time, especially in the presence of underlying hepatocyte growth and regeneration, (3) high dose vector targeting of non‐permissive tissue is required. (4) AAV vectors package single‐stranded DNA (ssDNA) and therefore require time‐consuming steps of complementary‐strand synthesis or recruitment for transduction. Repeated administration of AVV‐based gene therapy is not ideal due to the aforementioned application limitations. In addition to Ad and AAV‐targeted gene therapy for GSDI, one study used a lentiviral vector based on feline immunodeficiency virus (FIV).^111^ Compared to adeno‐associated viruses, lentiviral vectors are clinically attractive, including: (1) lentiviruses integrate transgene‐carrying viruses into cellular genes, providing long‐term expression even in actively dividing cells; (2) immune response to the vector components is not elicited because FIV long‐termina repeat activity is negligible. However, the copy number of the viral genome integrated into the cellular genome may vary considerably between experiments and the potential genotoxicity of integrated genomes; thus, the full potential of lentiviral vectors for GSDI gene therapy remains to be determined.

New therapeutic vehicles are currently being developed for an increasing number of indications. A new therapeutic concept that has recently been proposed with particular applicability to metabolic diseases is the combination of gene therapy and other therapeutic strategies. Prior to gene therapy, dietary regimens and pharmacological interventions can be very effective in preventing hypoglycemia and alleviating liver damage. The combination of dietary regimens or pharmacological treatment with gene therapy has the potential to enhance therapeutic outcomes through early treatment of complications and prolonged vector persistence. Significant challenges remain before gene therapy moves from proof‐of‐concept to preclinical studies and clinical trials; we need to further innovate technology in order to generate safer and more effective programmable vectors to promote clinical applications and ultimately improve child health.

## AUTHOR CONTRIBUTIONS

Jiamin Zhon, Jiaming Fan, Russell R. Reid, and Tong‐Chuan He conceived and designed the study. Jiamin Zhon, Yannian Gou, Piao Zhao, Xiangyu Dong, Meichun Guo, Aohua Li, Ailing Hao, Hue H. Luu, Tong‐Chuan He, Russell R. Reid, and Jiaming Fan drafted and revised the manuscript. All authors reviewed, edited, and approved the manuscript.

## CONFLICT OF INTEREST STATEMENT

Tong‐Chuan He, Hue H. Luu and Russell R. Reid are the members of *Pediatric Discovery* Editorial Board. They were excluded from all editorial decision‐making related to the acceptance of this article for publication. The remaining authors declare no conflict of interests.

## ETHICS STATEMENT

Not applicable.

## Data Availability

The data that support the findings of this study are available from the corresponding author upon reasonable request.

## References

[1] Molares‐Vila A , Corbalán‐Rivas A , Carnero‐Gregorio M , González‐Cespón JL , Rodríguez‐Cerdeira C . Biomarkers in glycogen storage diseases: an update. Int J Mol Sci. 2021;22(9):22. 10.3390/ijms22094381 PMC8122709 33922238

[2] Kishnani PS , Sun B , Koeberl DD . Gene therapy for glycogen storage diseases. Hum Mol Genet. 2019;28(R1):R31‐R41. 31227835 10.1093/hmg/ddz133 PMC6796997

[3] Sun Y , Qiang W , Wu R , et al. A glycogen storage disease type 1a patient with type 2 diabetes. BMC Med Genom. 2022;15(1):205. 10.1186/s12920-022-01344-3 PMC9516787 36167523

[4] Kaiser N , Gautschi M , Bosanska L , et al. Glycemic control and complications in glycogen storage disease type I: results from the Swiss registry. Mol Genet Metab. 2019;126(4):355‐361. 30846352 10.1016/j.ymgme.2019.02.008

[5] Yiu WH , Pan CJ , Allamarvdasht M , Kim SY , Chou JY . Glucose‐6‐phosphate transporter gene therapy corrects metabolic and myeloid abnormalities in glycogen storage disease type Ib mice. Gene Ther. 2007;14(3):219‐226. 17006547 10.1038/sj.gt.3302869 PMC2507880

[6] Kishnani PS , Austin SL , Abdenur JE , et al. Diagnosis and management of glycogen storage disease type I: a practice guideline of the American College of Medical Genetics and Genomics. Genet Med. 2014;16(11):e1‐e29. 25356975 10.1038/gim.2014.128

[7] Lee YM , Jun HS , Pan CJ , et al. Prevention of hepatocellular adenoma and correction of metabolic abnormalities in murine glycogen storage disease type Ia by gene therapy. Hepatology. 2012;56(5):1719‐1729. 22422504 10.1002/hep.25717 PMC3477505

[8] Gjorgjieva M , Raffin M , Duchampt A , et al. Progressive development of renal cysts in glycogen storage disease type I. Hum Mol Genet. 2016;25(17):3784‐3797. 27436577 10.1093/hmg/ddw224

[9] Luo X , Hall G , Li S , et al. Hepatorenal correction in murine glycogen storage disease type I with a double‐stranded adeno‐associated virus vector. Mol Ther. 2011;19(11):1961‐1970. 21730973 10.1038/mt.2011.126 PMC3222521

[10] Sim SW , Jang Y , Park TS , Park BC , Lee YM , Jun HS . Molecular mechanisms of aberrant neutrophil differentiation in glycogen storage disease type Ib. Cell Mol Life Sci. 2022;79(5):246. 35437689 10.1007/s00018-022-04267-5 PMC11071875

[11] Humbert M , Labrune P , Sitbon O , et al. Pulmonary arterial hypertension and type‐I glycogen‐storage disease: the serotonin hypothesis. Eur Respir J. 2002;20(1):59‐65. 12166582 10.1183/09031936.02.00258702

[12] Ohura T , Inoue CN , Abukawa D , et al. Progressive pulmonary hypertension: a fatal complication of type I glycogen storage disease. J Inherit Metab Dis. 1995;18(3):361‐362. 7474908 10.1007/BF00710433

[13] Ross KM , Ferrecchia IA , Dahlberg KR , Dambska M , Ryan PT , Weinstein DA . Dietary management of the glycogen storage diseases: evolution of treatment and ongoing controversies. Adv Nutr. 2020;11(2):439‐446. 31665208 10.1093/advances/nmz092 PMC7442342

[14] Della Pepa G , Vetrani C , Lupoli R , et al. Uncooked cornstarch for the prevention of hypoglycemic events. Crit Rev Food Sci Nutr. 2022;62(12):3250‐3263. 33455416 10.1080/10408398.2020.1864617

[15] High KA , Roncarolo MG . Gene therapy. N Engl J Med. 2019;381(5):455‐464. 31365802 10.1056/NEJMra1706910

[16] Gou Y , Weng Y , Chen Q , et al. Carboxymethyl chitosan prolongs adenovirus‐mediated expression of IL‐10 and ameliorates hepatic fibrosis in a mouse model. Bioeng Transl Med. 2022;7(3):e10306. 36176604 10.1002/btm2.10306 PMC9472002

[17] Lundstrom K . Viral vectors in gene therapy. Diseases. 2018;6(2):42. 29883422 10.3390/diseases6020042 PMC6023384

[18] Mendell JR , Al‐Zaidy SA , Rodino‐Klapac LR , et al. Current clinical applications of in vivo gene therapy with AAVs. Mol Ther. 2021;29(2):464‐488. 33309881 10.1016/j.ymthe.2020.12.007 PMC7854298

[19] Chou JY , Cho JH , Kim GY , Mansfield BC . Molecular biology and gene therapy for glycogen storage disease type Ib. J Inherit Metab Dis. 2018;41(6):1007‐1014. 29663270 10.1007/s10545-018-0180-5

[20] Derks TGJ , Rodriguez‐Buritica DF , Ahmad A , et al. Glycogen storage disease type Ia: current management options, burden and unmet needs. Nutrients. 2021;13(11):3828. 34836082 10.3390/nu13113828 PMC8621617

[21] Chou JY , Kim GY , Cho JH . Recent development and gene therapy for glycogen storage disease type Ia. Liver Res. 2017;1(3):174‐180. 29576889 10.1016/j.livres.2017.12.001 PMC5859325

[22] Chou JY , Mansfield BC . Mutations in the glucose‐6‐phosphatase‐alpha (G6PC) gene that cause type Ia glycogen storage disease. Hum Mutat. 2008;29(7):921‐930. 18449899 10.1002/humu.20772 PMC2475600

[23] Hemrika W , Renirie R , Dekker HL , Barnett P , Wever R . From phosphatases to vanadium peroxidases: a similar architecture of the active site. Proc Natl Acad Sci U S A. 1997;94(6):2145‐2149. 9122162 10.1073/pnas.94.6.2145 PMC20055

[24] Hemrika W , Wever R . A new model for the membrane topology of glucose‐6‐phosphatase: the enzyme involved in von Gierke disease. FEBS Lett. 1997;409(3):317‐319. 9224681 10.1016/s0014-5793(97)00530-9

[25] Sim SW , Weinstein DA , Lee YM , Jun HS . Glycogen storage disease type Ib: role of glucose‐6‐phosphate transporter in cell metabolism and function. FEBS Lett. 2020;594(1):3‐18. 31705665 10.1002/1873-3468.13666

[26] Chou JY , Jun HS , Mansfield BC . Glycogen storage disease type I and G6Pase‐β deficiency: etiology and therapy. Nat Rev Endocrinol. 2010;6(12):676‐688. 20975743 10.1038/nrendo.2010.189 PMC4178929

[27] Lee YM , Conlon TJ , Specht A , et al. Long‐term safety and efficacy of AAV gene therapy in the canine model of glycogen storage disease type Ia. J Inherit Metab Dis. 2018;41(6):977‐984. 29802554 10.1007/s10545-018-0199-7

[28] Mathis T , Poms M , Köfeler H , et al. Untargeted plasma metabolomics identifies broad metabolic perturbations in glycogen storage disease type I. J Inherit Metab Dis. 2022;45(2):235‐247. 34671989 10.1002/jimd.12451 PMC9299190

[29] Lee PJ . Glycogen storage disease type I: pathophysiology of liver adenomas. Eur J Pediatr. 2002;161(Suppl 1):S46‐S49. 12373570 10.1007/s00431-002-1002-0

[30] Kim GY , Kwon JH , Cho JH , Zhang L , Mansfield BC , Chou JY . Downregulation of pathways implicated in liver inflammation and tumorigenesis of glycogen storage disease type Ia mice receiving gene therapy. Hum Mol Genet. 2017;26(10):1890‐1899. 28334808 10.1093/hmg/ddx097 PMC6075378

[31] Kim GY , Lee YM , Cho JH , et al. Mice expressing reduced levels of hepatic glucose‐6‐phosphatase‐α activity do not develop age‐related insulin resistance or obesity. Hum Mol Genet. 2015;24(18):5115‐5125. 26089201 10.1093/hmg/ddv230 PMC4550813

[32] Lee YM , Kim GY , Pan CJ , Mansfield BC , Chou JY . Minimal hepatic glucose‐6‐phosphatase‐α activity required to sustain survival and prevent hepatocellular adenoma formation in murine glycogen storage disease type Ia. Mol Genet Metab Rep. 2015;3:28‐32. 26937391 10.1016/j.ymgmr.2015.03.001 PMC4750588

[33] Kwon JH , Lee YM , Cho JH , et al. Liver‐directed gene therapy for murine glycogen storage disease type Ib. Hum Mol Genet. 2017;26(22):4395‐4405. 28973635 10.1093/hmg/ddx325 PMC5886224

[34] Rutten MGS , Derks TGJ , Huijkman NCA , et al. Modeling phenotypic heterogeneity of glycogen storage disease type 1a liver disease in mice by somatic CRISPR/CRISPR‐associated protein 9‐mediated gene editing. Hepatology. 2021;74(5):2491‐2507. 34157136 10.1002/hep.32022 PMC8597008

[35] Farah BL , Landau DJ , Wu Y , et al. Renal endoplasmic reticulum stress is coupled to impaired autophagy in a mouse model of GSD Ia. Mol Genet Metab. 2017;122(3):95‐98. 28888852 10.1016/j.ymgme.2017.08.013 PMC5722666

[36] Gjorgjieva M , Monteillet L , Calderaro J , Mithieux G , Rajas F . Polycystic kidney features of the renal pathology in glycogen storage disease type I: possible evolution to renal neoplasia. J Inherit Metab Dis. 2018;41(6):955‐963. 29869165 10.1007/s10545-018-0207-y

[37] Baker L , Dahlem S , Goldfarb S , et al. Hyperfiltration and renal disease in glycogen storage disease, type I. Kidney Int. 1989;35(6):1345‐1350. 2671467 10.1038/ki.1989.133

[38] Martens DH , Rake JP , Navis G , Fidler V , van Dael CM , Smit GP . Renal function in glycogen storage disease type I, natural course, and renopreservative effects of ACE inhibition. Clin J Am Soc Nephrol. 2009;4(11):1741‐1746. 19808227 10.2215/CJN.00050109 PMC2774963

[39] Koeberl DD , Pinto C , Sun B , et al. AAV vector‐mediated reversal of hypoglycemia in canine and murine glycogen storage disease type Ia. Mol Ther. 2008;16(4):665‐672. 18362924 10.1038/mt.2008.15

[40] Clar J , Gri B , Calderaro J , et al. Targeted deletion of kidney glucose‐6 phosphatase leads to nephropathy. Kidney Int. 2014;86(4):747‐756. 24717294 10.1038/ki.2014.102 PMC5678048

[41] Yiu WH , Mead PA , Jun HS , Mansfield BC , Chou JY . Oxidative stress mediates nephropathy in type Ia glycogen storage disease. Lab Invest. 2010;90(4):620‐629. 20195241 10.1038/labinvest.2010.38 PMC3078689

[42] Weinstein DA , Somers MJ , Wolfsdorf JI . Decreased urinary citrate excretion in type 1a glycogen storage disease. J Pediatr. 2001;138(3):378‐382. 11241046 10.1067/mpd.2001.111322

[43] Halligan RK , Dalton RN , Turner C , Lewis KA , Mundy HR . Understanding the role of SGLT2 inhibitors in glycogen storage disease type Ib: the experience of one UK centre. Orphanet J Rare Dis. 2022;17(1):195. 35549996 10.1186/s13023-022-02345-2 PMC9096769

[44] Wortmann SB , Van Hove JLK , Derks TGJ , et al. Treating neutropenia and neutrophil dysfunction in glycogen storage disease type Ib with an SGLT2 inhibitor. Blood. 2020;136(9):1033‐1043. 32294159 10.1182/blood.2019004465 PMC7530374

[45] Melis D , Pivonello R , Parenti G , et al. Increased prevalence of thyroid autoimmunity and hypothyroidism in patients with glycogen storage disease type I. J Pediatr. 2007;150(3):300‐305.e1. 17307551 10.1016/j.jpeds.2006.11.056

[46] Kuijpers TW , Maianski NA , Tool AT , et al. Apoptotic neutrophils in the circulation of patients with glycogen storage disease type 1b (GSD1b). Blood. 2003;101(12):5021‐5024. 12576310 10.1182/blood-2002-10-3128

[47] Letkemann R , Wittkowski H , Antonopoulos A , et al. Partial correction of neutrophil dysfunction by oral galactose therapy in glycogen storage disease type Ib. Int Immunopharmacol. 2017;44:216‐225. 28126686 10.1016/j.intimp.2017.01.020

[48] Veiga‐da‐Cunha M , Chevalier N , Stephenne X , et al. Failure to eliminate a phosphorylated glucose analog leads to neutropenia in patients with G6PT and G6PC3 deficiency. Proc Natl Acad Sci U S A. 2019;116(4):1241‐1250. 30626647 10.1073/pnas.1816143116 PMC6347702

[49] Starzl TE , Putnam CW . Portal diversion. Treatment for glycogen storage disease and hyperlipemia. JAMA. 1975;233(9):955‐957. 168416 10.1001/jama.233.9.955 PMC2975524

[50] Greene HL , Slonim AE , O'Neill JA, Jr. , Burr IM . Continuous nocturnal intragastric feeding for management of type 1 glycogen‐storage disease. N Engl J Med. 1976;294(8):423‐425. 813144 10.1056/NEJM197602192940805

[51] Heller S , Worona L , Consuelo A . Nutritional therapy for glycogen storage diseases. J Pediatr Gastroenterol Nutr. 2008;47(Suppl 1):S15‐S21. 18667910 10.1097/MPG.0b013e3181818ea5

[52] Schwenk WF , Haymond MW . Optimal rate of enteral glucose administration in children with glycogen storage disease type I. N Engl J Med. 1986;314(11):682‐685. 3081806 10.1056/NEJM198603133141104

[53] Wolfsdorf JI , Crigler JF, Jr. Effect of continuous glucose therapy begun in infancy on the long‐term clinical course of patients with type I glycogen storage disease. J Pediatr Gastroenterol Nutr. 1999;29(2):136‐143. 10435649 10.1097/00005176-199908000-00008

[54] Okechuku GO , Shoemaker LR , Dambska M , Brown LM , Mathew J , Weinstein DA . Tight metabolic control plus ACE inhibitor therapy improves GSD I nephropathy. J Inherit Metab Dis. 2017;40(5):703‐708. 28612263 10.1007/s10545-017-0054-2

[55] Melis D , Parenti G , Gatti R , et al. Efficacy of ACE‐inhibitor therapy on renal disease in glycogen storage disease type 1: a multicentre retrospective study. Clin Endocrinol. 2005;63(1):19‐25. 10.1111/j.1365-2265.2005.02292.x 15963056

[56] Aoun B , Sanjad S , Degheili JA , Barhoumi A , Bassyouni A , Karam PE . Kidney and metabolic phenotypes in glycogen storage disease type‐I patients. Front Pediatr. 2020;8:591. 33042926 10.3389/fped.2020.00591 PMC7518374

[57] Li AM , Thyagu S , Maze D , et al. Prolonged granulocyte colony stimulating factor use in glycogen storage disease type 1b associated with acute myeloid leukemia and with shortened telomere length. Pediatr Hematol Oncol. 2018;35(1):45‐51. 29652549 10.1080/08880018.2018.1440675

[58] Ishiguro A , Nakahata T , Shimbo T , et al. Improvement of neutropenia and neutrophil dysfunction by granulocyte colony‐stimulating factor in a patient with glycogen storage disease type Ib. Eur J Pediatr. 1993;152(1):18‐20. 7680314 10.1007/BF02072510

[59] Alsultan A , Sokol RJ , Lovell MA , Thurman G , Ambruso DR . Long term G‐CSF‐induced remission of ulcerative colitis‐like inflammatory bowel disease in a patient with glycogen storage disease Ib and evaluation of associated neutrophil function. Pediatr Blood Cancer. 2010;55(7):1410‐1413. 20830779 10.1002/pbc.22706

[60] Dale DC , Bolyard AA , Marrero T , et al. Neutropenia in glycogen storage disease Ib: outcomes for patients treated with granulocyte colony‐stimulating factor. Curr Opin Hematol. 2019;26(1):16‐21. 30451720 10.1097/MOH.0000000000000474 PMC7000169

[61] Melis D , Della Casa R , Parini R , et al. Vitamin E supplementation improves neutropenia and reduces the frequency of infections in patients with glycogen storage disease type 1b. Eur J Pediatr. 2009;168(9):1069‐1074. 19066956 10.1007/s00431-008-0889-5

[62] Melis D , Minopoli G , Balivo F , et al. Vitamin E improves clinical outcome of patients affected by glycogen storage disease type Ib. JIMD Rep. 2016;25:39‐45. 26122627 10.1007/8904_2015_461 PMC5059207

[63] Khalaf D , Bell H , Dale D , et al. A case of secondary acute myeloid leukemia on a background of glycogen storage disease with chronic neutropenia treated with granulocyte colony stimulating factor. JIMD Rep. 2019;49(1):37‐42. 31788408 10.1002/jmd2.12069 PMC6875697

[64] Grünert SC , Elling R , Maag B , et al. Improved inflammatory bowel disease, wound healing and normal oxidative burst under treatment with empagliflozin in glycogen storage disease type Ib. Orphanet J Rare Dis. 2020;15(1):218. 32838757 10.1186/s13023-020-01503-8 PMC7446198

[65] Grünert SC , Derks TGJ , Adrian K , et al. Efficacy and safety of empagliflozin in glycogen storage disease type Ib: data from an international questionnaire. Genet Med. 2022;24(8):1781‐1788. 35503103 10.1016/j.gim.2022.04.001

[66] Rossi A , Miele E , Fecarotta S , et al. Crohn disease‐like enterocolitis remission after empagliflozin treatment in a child with glycogen storage disease type Ib: a case report. Ital J Pediatr. 2021;47(1):149. 34215305 10.1186/s13052-021-01100-w PMC8254289

[67] Hexner‐Erlichman Z , Veiga‐da‐Cunha M , Zehavi Y , et al. Favorable outcome of empagliflozin treatment in two pediatric glycogen storage disease type 1b patients. Front Pediatr. 2022;10:1071464. 36507137 10.3389/fped.2022.1071464 PMC9727171

[68] Grünert SC , Rosenbaum‐Fabian S , Schumann A , et al. Two successful pregnancies and first use of empagliflozin during pregnancy in glycogen storage disease type Ib. JIMD Rep. 2022;63(4):303‐308. 35822091 10.1002/jmd2.12295 PMC9259388

[69] Zhou J , Waskowicz LR , Lim A , et al. A liver‐specific thyromimetic, VK2809, decreases hepatosteatosis in glycogen storage disease type Ia. Thyroid. 2019;29(8):1158‐1167. 31337282 10.1089/thy.2019.0007 PMC6707038

[70] Yavarow ZA , Kang HR , Waskowicz LR , et al. Fenofibrate rapidly decreases hepatic lipid and glycogen storage in neonatal mice with glycogen storage disease type Ia. Hum Mol Genet. 2020;29(2):286‐294. 31816064 10.1093/hmg/ddz290 PMC7003036

[71] Waskowicz LR , Zhou J , Landau DJ , et al. Bezafibrate induces autophagy and improves hepatic lipid metabolism in glycogen storage disease type Ia. Hum Mol Genet. 2019;28(1):143‐154. 30256948 10.1093/hmg/ddy343 PMC6298237

[72] Monteillet L , Gjorgjieva M , Silva M , et al. Intracellular lipids are an independent cause of liver injury and chronic kidney disease in non alcoholic fatty liver disease‐like context. Mol Metab. 2018;16:100‐115. 30100243 10.1016/j.molmet.2018.07.006 PMC6157648

[73] Farah BL , Landau DJ , Sinha RA , et al. Induction of autophagy improves hepatic lipid metabolism in glucose‐6‐phosphatase deficiency. J Hepatol. 2016;64(2):370‐379. 26462884 10.1016/j.jhep.2015.10.008

[74] Zingone A , Hiraiwa H , Pan CJ , et al. Correction of glycogen storage disease type 1a in a mouse model by gene therapy. J Biol Chem. 2000;275(2):828‐832. 10625614 10.1074/jbc.275.2.828

[75] Chou JY , Zingone A , Pan CJ . Adenovirus‐mediated gene therapy in a mouse model of glycogen storage disease type 1a. Eur J Pediatr. 2002;161(Suppl 1):S56‐S61. 12373573 10.1007/s00431-002-1005-x

[76] Sun MS , Pan CJ , Shieh JJ , et al. Sustained hepatic and renal glucose‐6‐phosphatase expression corrects glycogen storage disease type Ia in mice. Hum Mol Genet. 2002;11(18):2155‐2164. 12189168 10.1093/hmg/11.18.2155

[77] Koeberl DD , Sun B , Bird A , Chen YT , Oka K , Chan L . Efficacy of helper‐dependent adenovirus vector‐mediated gene therapy in murine glycogen storage disease type Ia. Mol Ther. 2007;15(7):1253‐1258. 17505475 10.1038/sj.mt.6300188

[78] Crane B , Luo X , Demaster A , et al. Rescue administration of a helper‐dependent adenovirus vector with long‐term efficacy in dogs with glycogen storage disease type Ia. Gene Ther. 2012;19(4):443‐452. 21654821 10.1038/gt.2011.86

[79] Beaty RM , Jackson M , Peterson D , et al. Delivery of glucose‐6‐phosphatase in a canine model for glycogen storage disease, type Ia, with adeno‐associated virus (AAV) vectors. Gene Ther. 2002;9(15):1015‐1022. 12101432 10.1038/sj.gt.3301728

[80] Michelfelder S , Trepel M . Adeno‐associated viral vectors and their redirection to cell‐type specific receptors. Adv Genet. 2009;67:29‐60. 19914449 10.1016/S0065-2660(09)67002-4

[81] Gao GP , Alvira MR , Wang L , Calcedo R , Johnston J , Wilson JM . Novel adeno‐associated viruses from rhesus monkeys as vectors for human gene therapy. Proc Natl Acad Sci U S A. 2002;99(18):11854‐11859. 12192090 10.1073/pnas.182412299 PMC129358

[82] Koeberl DD , Sun BD , Damodaran TV , et al. Early, sustained efficacy of adeno‐associated virus vector‐mediated gene therapy in glycogen storage disease type Ia. Gene Ther. 2006;13(17):1281‐1289. 16672983 10.1038/sj.gt.3302774

[83] Yiu WH , Lee YM , Peng WT , et al. Complete normalization of hepatic G6PC deficiency in murine glycogen storage disease type Ia using gene therapy. Mol Ther. 2010;18(6):1076‐1084. 20389290 10.1038/mt.2010.64 PMC2889730

[84] Demaster A , Luo X , Curtis S , et al. Long‐term efficacy following readministration of an adeno‐associated virus vector in dogs with glycogen storage disease type Ia. Hum Gene Ther. 2012;23(4):407‐418. 22185325 10.1089/hum.2011.106 PMC4047999

[85] Wang L . Adeno‐associated virus gene therapy prevents hepatocellular adenoma in murine model of glycogen storage disease type Ia. Hepatology. 2012;56(5):1593‐1595. 22706804 10.1002/hep.25894

[86] Brooks ED , Landau DJ , Everitt JI , et al. Long‐term complications of glycogen storage disease type Ia in the canine model treated with gene replacement therapy. J Inherit Metab Dis. 2018;41(6):965‐976. 30043186 10.1007/s10545-018-0223-y PMC6328337

[87] Kim GY , Lee YM , Kwon JH , et al. Glycogen storage disease type Ia mice with less than 2% of normal hepatic glucose‐6‐phosphatase‐α activity restored are at risk of developing hepatic tumors. Mol Genet Metab. 2017;120(3):229‐234. 28096054 10.1016/j.ymgme.2017.01.003 PMC5346453

[88] Zhang L , Lee C , Arnaoutova I , et al. Gene therapy using a novel G6PC‐S298C variant enhances the long‐term efficacy for treating glycogen storage disease type Ia. Biochem Biophys Res Commun. 2020;527(3):824‐830. 32430177 10.1016/j.bbrc.2020.04.124 PMC7309276

[89] Cao J , Choi M , Guadagnin E , et al. mRNA therapy restores euglycemia and prevents liver tumors in murine model of glycogen storage disease. Nat Commun. 2021;12(1):3090. 34035281 10.1038/s41467-021-23318-2 PMC8149455

[90] Zhang L , Cho JH , Arnaoutova I , Mansfield BC , Chou JY . An evolutionary approach to optimizing glucose‐6‐phosphatase‐α enzymatic activity for gene therapy of glycogen storage disease type Ia. J Inherit Metab Dis. 2019;42(3):470‐479. 30714174 10.1002/jimd.12069 PMC6483894

[91] Penaud‐Budloo M , Le Guiner C , Nowrouzi A , et al. Adeno‐associated virus vector genomes persist as episomal chromatin in primate muscle. J Virol. 2008;82(16):7875‐7885. 18524821 10.1128/JVI.00649-08 PMC2519600

[92] Miller DG , Petek LM , Russell DW . Adeno‐associated virus vectors integrate at chromosome breakage sites. Nat Genet. 2004;36(7):767‐773. 15208627 10.1038/ng1380

[93] Landau DJ , Brooks ED , Perez‐Pinera P , et al. In vivo zinc finger nuclease‐mediated targeted integration of a glucose‐6‐phosphatase transgene promotes survival in mice with glycogen storage disease type IA. Mol Ther. 2016;24(4):697‐706. 26865405 10.1038/mt.2016.35 PMC4886939

[94] Perez‐Pinera P , Ousterout DG , Brown MT , Gersbach CA . Gene targeting to the ROSA26 locus directed by engineered zinc finger nucleases. Nucleic Acids Res. 2012;40(8):3741‐3752. 22169954 10.1093/nar/gkr1214 PMC3333879

[95] Hermann M , Maeder ML , Rector K , et al. Evaluation of OPEN zinc finger nucleases for direct gene targeting of the ROSA26 locus in mouse embryos. PLoS One. 2012;7(9):e41796. 22970113 10.1371/journal.pone.0041796 PMC3435328

[96] Kang HR , Gjorgjieva M , Smith SN , et al. Pathogenesis of hepatic tumors following gene therapy in murine and canine models of glycogen storage disease. Mol Ther Methods Clin Dev. 2019;15:383‐391. 31890731 10.1016/j.omtm.2019.10.016 PMC6909089

[97] Parikh NS , Ahlawat R . Glycogen Storage Disease Type I. StatPearls. StatPearls Publishing Copyright © 2022, StatPearls Publishing LLC.; 2022. 30480935

[98] Ross KM , Brown LM , Corrado MM , et al. Safety and efficacy of chronic extended release cornstarch therapy for glycogen storage disease type I. JIMD Rep. 2016;26:85‐90. 26303612 10.1007/8904_2015_488 PMC4864714

[99] Duran J , Gruart A , García‐Rocha M , Delgado‐García JM , Guinovart JJ . Glycogen accumulation underlies neurodegeneration and autophagy impairment in Lafora disease. Hum Mol Genet. 2014;23(12):3147‐3156. 24452334 10.1093/hmg/ddu024

[100] Raben N , Hill V , Shea L , et al. Suppression of autophagy in skeletal muscle uncovers the accumulation of ubiquitinated proteins and their potential role in muscle damage in Pompe disease. Hum Mol Genet. 2008;17(24):3897‐3908. 18782848 10.1093/hmg/ddn292 PMC2638578

[101] Singh R , Kaushik S , Wang Y , et al. Autophagy regulates lipid metabolism. Nature. 2009;458(7242):1131‐1135. 19339967 10.1038/nature07976 PMC2676208

[102] Kang HR , Waskowicz L , Seifts AM , Landau DJ , Young SP , Koeberl DD . Bezafibrate enhances AAV vector‐mediated genome editing in glycogen storage disease type Ia. Mol Ther Methods Clin Dev. 2019;13:265‐273. 30859111 10.1016/j.omtm.2019.02.002 PMC6395830

[103] Zhou J , Singh BK , Ho JP , et al. MED1 mediator subunit is a key regulator of hepatic autophagy and lipid metabolism. Autophagy. 2021;17(12):4043‐4061. 33734012 10.1080/15548627.2021.1899691 PMC8726716

[104] Mundy HR , Lee PJ . Glycogenosis type I and diabetes mellitus: a common mechanism for renal dysfunction? Med Hypotheses. 2002;59(1):110‐114. 12160694 10.1016/s0306-9877(02)00199-8

[105] López‐Hernández FJ , López‐Novoa JM . Role of TGF‐β in chronic kidney disease: an integration of tubular, glomerular and vascular effects. Cell Tissue Res. 2012;347(1):141‐154. 22105921 10.1007/s00441-011-1275-6

[106] Loeffler I , Wolf G . Transforming growth factor‐β and the progression of renal disease. Nephrol Dial Transpl. 2014;29(Suppl 1):i37‐i45. 10.1093/ndt/gft267 24030832

[107] Ghosh A , Allamarvdasht M , Pan CJ , et al. Long‐term correction of murine glycogen storage disease type Ia by recombinant adeno‐associated virus‐1‐mediated gene transfer. Gene Ther. 2006;13(4):321‐329. 16195703 10.1038/sj.gt.3302650

[108] Yiu WH , Pan CJ , Mead PA , Starost MF , Mansfield BC , Chou JY . Normoglycemia alone is insufficient to prevent long‐term complications of hepatocellular adenoma in glycogen storage disease type Ib mice. J Hepatol. 2009;51(5):909‐917. 19376605 10.1016/j.jhep.2008.11.026 PMC2762018

[109] Lee YM , Pan CJ , Koeberl DD , Mansfield BC , Chou JY . The upstream enhancer elements of the G6PC promoter are critical for optimal G6PC expression in murine glycogen storage disease type Ia. Mol Genet Metab. 2013;110(3):275‐280. 23856420 10.1016/j.ymgme.2013.06.014 PMC3898731

[110] Wang D , Tai PWL , Gao G . Adeno‐associated virus vector as a platform for gene therapy delivery. Nat Rev Drug Discov. 2019;18(5):358‐378. 30710128 10.1038/s41573-019-0012-9 PMC6927556

[111] Grinshpun A , Condiotti R , Waddington SN , et al. Neonatal gene therapy of glycogen storage disease type Ia using a feline immunodeficiency virus‐based vector. Mol Ther. 2010;18(9):1592‐1598. 20571544 10.1038/mt.2010.119 PMC2956916

